# A Curious Case of Intestinal Diaphragm Disease Unmasked by Perforation of a Duodenal Ulcer

**DOI:** 10.1155/2017/5048345

**Published:** 2017-03-27

**Authors:** Mairéad McNally, Ion Cretu

**Affiliations:** Naas General Hospital Affiliated with Trinity College Dublin, Naas, County Kildare, Ireland

## Abstract

Nonsteroidal anti-inflammatory drugs are a common cause of intestinal injury. A variety of NSAID-induced injuries may occur including ulcers, erosions, colitis, strictures, and diaphragm disease. Diaphragm disease refers to the development of multiple thin, concentric, stenosing strictures in the intestine. Strictures occur most often in the midintestine and are thought to be pathognomonic of NSAID damage. They can lead to intermittent or complete bowel obstruction. Diagnosis may be elusive as there is nothing specific about NSAID-induced injury at endoscopy and histology is also nonspecific. Even at laparotomy, the diagnosis of diaphragm disease may be missed as the serosa may appear normal and strictures can be difficult to palpate. While most NSAID-induced lesions tend to resolve quickly following withdrawal of the offending drug, diaphragm-like strictures usually require intervention such as stricturoplasty or surgical resection of the involved segment of bowel. Here we report the case of a 60-year-old male patient who presented with iron deficiency anaemia and recurrent subacute bowel obstruction. Following endoscopy and repeated CT scanning of his abdomen, he was diagnosed with Crohn's disease. He was treated with 5-ASAs and immune suppression until a perforated duodenal ulcer resulted in emergency laparotomy and the subsequent discovery of multiple intestinal diaphragms attributable to long-standing NSAID use.

## 1. Case

In June 2010, a 60-year-old male patient presented to clinic for investigation of iron deficiency anaemia and unintentional weight loss. He reported mild heartburn. He had a background medical history of depression, hypertension, hypercholesterolaemia, osteoarthritis, and previous right knee meniscectomy. He admitted to minimal alcohol intake and was an ex-smoker. Regular medications were combination of Lisinopril 20 mg and hydrochlorothiazide 12.5 mg od, Fluoxetine 20 mg od, and Atorvastatin 10 mg od. Relevant laboratory investigations on initial assessment included iron of 4.1 *μ*mol/L (normal 14–31), Hb 9.7 g/dL (normal 13.0–18.0), and transferrin saturation 6.4% (normal 30–40). B12 and folate levels were normal. OGD revealed antral gastritis and full colonoscopy to the caecum was normal. Coeliac antibodies were negative and biopsies confirmed histologically normal colonic and duodenal mucosa with preservation of normal villous architecture. He commenced treatment with oral iron supplementation and was lost to follow up until he was admitted to the hospital with severe abdominal pain in March 2011.

**Figure 1:**
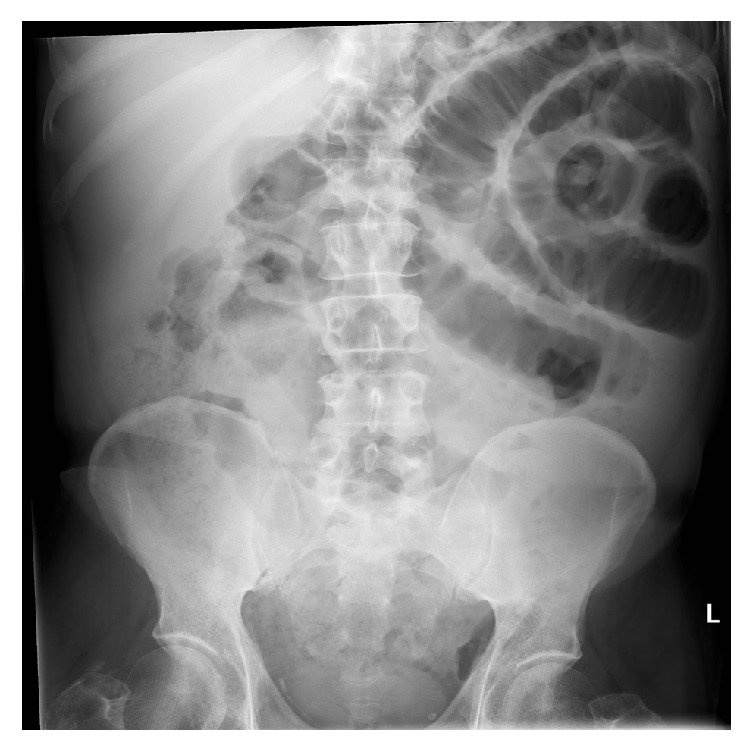
Plain film of abdomen showing dilated small bowel loops. March 2011.

In 2011, bloods showed an elevated white cell count of 16.5 × 10^9^/L (normal 4.0–11.0), neutrophil count of 12.7 × 10^9^/L (normal 2.0–7.5), and a platelet count of 638 × 10^9^/L (normal 150–400). Hb was 13.6 g/dL with normal MCV and MCH values. CRP was 25 mg/dL (normal < 5). Liver enzymes were within normal range with an albumin level of 39 g/L (normal 34–38). He had an acute kidney injury with urea of 16.4 mmol/L (normal 2.1–7.1) and creatinine 144 *μ*mol/L (normal 62–106) on a background of previously normal renal function. PFA showed dilated small bowel loops. See Figure 1 [1].

**Figure 2:**
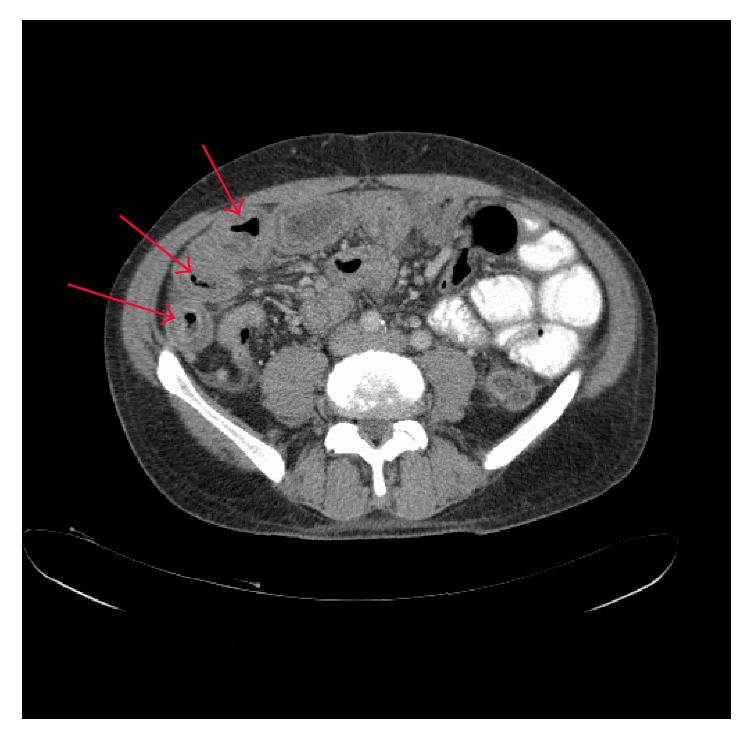
CT abdomen axial view showing thickening of the small bowel wall as indicated by the arrows. March 2011.

CT abdomen and pelvis revealed several considerably thickened proximal ileal loops with mildly prominent jejunal loops and small volume ascites suggestive of inflammatory bowel disease. See Figure 2 [1].

His symptoms and blood work settled with conservative management and he was discharged for outpatient follow-up. He continued to take oral iron supplementation.

In April 2012, small bowel follow through showed several dilated jejunal loops measuring up to 6 cm in diameter with regular thickening of the small bowel folds. The terminal ileum was not optimally visualised but appeared normal. Crohn's disease was considered a possible cause of the presentation although the reporting radiologist suggested a differential diagnosis of Whipple's disease or giardiasis. The patient reported vague intermittent abdominal discomfort then but did not commence any treatment.

In July 2013, he proceeded to anterograde double balloon enteroscopy. The procedure was completed successfully to the jejunum and showed that the entire duodenum had duodenitis and definite scalloping of the mucosa. The patient was prescribed a proton pump inhibitor. He was monitored in clinic and continued to report episodic abdominal pain. He was given intravenous iron for persistent iron deficiency anaemia.

Capsule endoscopy was ordered but before this was performed, the patient represented to hospital in May 2014 with a subacute bowel obstruction. He had mildly elevated inflammatory markers (WCC 15.5 Plts 517 CRP 60) and CT abdomen and pelvis noted distal small bowel obstruction.

He settled with conservative management and was labelled with a radiological diagnosis of small bowel Crohn's disease. He commenced on a reducing dose of oral prednisolone and regular Mesalazine 2 g bd. Three months later Azathioprine 50 mg od was introduced to the patient's regimen.

In February 2015, the patient underwent repeat endoscopy as his haemoglobin dropped to 7.9 g/dL. His iron level dropped to 2 *μ*mol/L, transferrin saturations measured 4%, and serum ferritin was 3.8 *μ*g/L (normal 23.0–393.0). OGD showed mild oesophagitis and linear gastritis.

Full colonoscopy to the terminal ileum was entirely normal, with normal colonic and small bowel mucosa on histology. The diagnosis of Crohn's disease was called into question. Mesalazine and Azathioprine were discontinued but the patient experienced a flare of symptom 2 months later and, at his request, his Crohn's medications were reintroduced.

**Figure 3:**
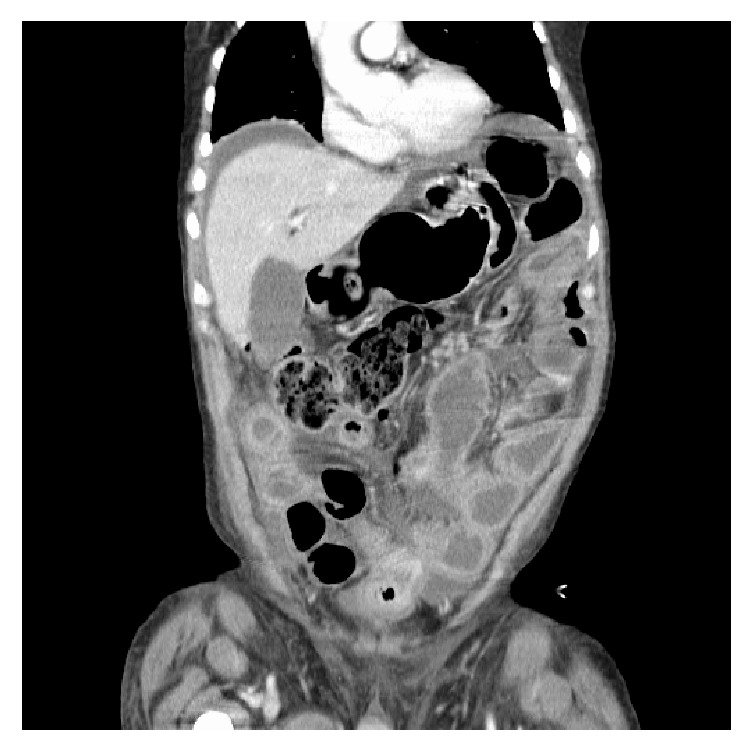
CT abdomen coronal view after IV contrast. There is significant dilatation noted in the loops of the small intestines and some of them showed diffuse wall thickening. There is a large amount of free fluid noted in the abdomen and pelvis. There is free air noted in the abdomen and pelvis indicating bowel perforation. May 2016.

His third admission to hospital occurred in May 2016 when he presented to the emergency department with severe abdominal pain and distension. CT scan noted significant dilatation of small bowel loops with diffuse wall thickening. There was a large amount of free fluid noted in the abdomen and pelvis and free air in the abdomen indicated a bowel perforation. See Figure 3 [1].

He proceeded to laparotomy where he was found to have a large perforated duodenal ulcer involving more than 50% of the circumference of the duodenal wall. Surgeons found also 15 circumferential strictures along the ileum at laparotomy. The strictures were separated by macroscopically normal small bowel giving the pathognomonic appearance of intestinal diaphragm disease. Surgeons, who were unable to photograph the findings, described a “string of sausages” appearance to the bowel. Multiple stricturoplasties were performed.

8 days postoperatively the patient deteriorated due to a leak from the site of previous stricturoplasty and surgical resection of a segment of small bowel was performed with formation of a defunctioning ileostomy. Most of the segments of small bowel with intestinal diaphragms managed by stricturoplasty remained in situ. A 16.5 cm × 5 cm × 4 cm segment of small bowel was sent for pathological examination. There was extensive white exudate present on the external surface. The mucosal surface appeared strictured and flattened with a thickened bowel wall. Histological review of representative sections from the small bowel demonstrated circumferential mucosal ulceration and transmural inflammation. No features of Crohn's disease were identified.

A thorough review of the case uncovered a long-standing history of NSAID use in the past and this confirmed the diagnosis of NSAID-induced diaphragm disease.

The patient admitted to a history of Diclofenac use PRN for 2 years prior to his initial presentation. He took Diclofenac 50 mg intermittently for knee and back pain due to osteoarthritis and stopped taking the drug approximately 3 months before presenting to our services. He was not on a regular proton pump inhibitor (PPI). Although Diclofenac is only available on prescription it did not come to light as part of his medical history. Since the patient had taken it in the past and did not consider it relevant he did not report previous use. After presentation to our services, he did not receive any additional prescriptions for Diclofenac but used over-the-counter ibuprofen intermittently for the next 3 years.

In retrospect, NSAID use accounted for the finding of acute kidney injury in 2011 and gastritis noted at endoscopy. The patient was discharged home one month postoperatively. All Crohn's medications and NSAIDs were discontinued and he remains well.

## 2. Discussion

Diaphragm disease, first described by Lang et al. in 1988 [2], is an uncommon but important cause of recurrent small bowel obstruction. It involves the formation of multiple thin, concentric, stenosing strictures in the intestine. While the disease has a distinctive macroscopic appearance, it is associated with an array of histological features. When first describing the disease, Lang et al. [2] noted that the diaphragmatic lesions demonstrated a focus of submucosal fibrosis and found increased numbers of neutrophil polymorphs, lymphocytes, eosinophils, and plasma cells in the overlying abnormal mucosa. Intestinal mucosa between diaphragms was noted to be almost entirely normal. Additional findings noted in later studies include villous atrophy [3] and ulceration of the mucosa at the tip of diaphragms [4].

The pathogenesis of the disease is unclear but it has been linked with long term NSAID use [5–7]. It has been hypothesised that NSAID use leads to a reduction in mucosal synthesis of prostaglandins causing disruption of the mucosal integrity making the mucosa more vulnerable to bacteria or toxins [2].

In this case report, it is likely that the patient's initial presentation with iron deficiency anaemia was related to gastritis and associated small bowel inflammation due to NSAID use. It has been shown that NSAIDs can induce injuries in the small bowel even in patients with minimal or nonexistent lesions in the stomach [8]. We suspect that our patient had small bowel inflammation at his first presentation and that areas of inflammation ultimately evolved over years to develop into diaphragm disease. Many [2, 4, 9], but not all, cases of intestinal diaphragm disease in the literature are associated with iron deficiency anaemia. As the effects of NSAIDs on the gut are widespread and variable, diaphragm disease may be viewed as part of a spectrum of intestinal mucosal damage related to NSAID use with associated anaemia arising mainly from occult bleeding in areas of NSAID-induced inflammation. It is estimated that diaphragm disease occurs in 2% of chronic NSAID users and may cause subacute bowel obstruction in a small subset of these patients [10]. In keeping with this view of diaphragm disease, it is interesting to note that our patient showed evidence of gastritis on his first OGD and ultimately his diaphragm disease was unmasked by significant duodenitis leading to perforation of a duodenal ulcer. It is not known how long it takes from exposure to NSAID to the development of intestinal diaphragms although we know that the effects of NSAIDs may persist for long periods even after the drug has been discontinued [11]. It is unlikely that the lack of PPI in our patient's regimen contributed to the development of diaphragm disease as it is now thought that PPIs do not provide protection against NSAID-induced damage to the small intestine [12].

Although any small intestinal pathology may be associated with small bowel bacterial overgrowth and resultant malabsorption, Lang et al. [2] observed that malabsorption tends not to be a predominant feature in cases of diaphragm disease. De Petris and Lopez [4] published a study of 10 cases of diaphragm disease of the small intestine from a single institution in which they identified 9 cases with iron deficiency anaemia but only a single case associated with a protein-losing enteropathy. Similarly, our patient did not develop hypoalbuminaemia at any time. To the authors' knowledge there are no reports of diaphragm disease in the literature documenting micronutrient deficiencies.

Diaphragm disease can mimic inflammatory bowel disease and poses a diagnostic challenge. Radiological diagnosis is difficult as diaphragms tend to be thin and do not distort the bowel wall. Small bowel studies with luminal contrast are neither sensitive nor specific for detecting intestinal diaphragms [10]. Capsule endoscopy is useful in identifying NSAID-induced small bowel damage [13, 14] and may aid in the diagnosis of diaphragm disease [15] but stenosing strictures can result in restricted passage of the capsule. Double balloon endoscopy (DBE) also has role to play in identifying drug-induced injuries in the small bowel including diaphragm disease [8, 16] and some studies have shown dilatation at DBE to be an effective treatment for diaphragm disease [16]. In some cases of diaphragm disease however, strictures are multiple and laparotomy with stricturoplasty or bowel resection may be required for effective treatment.

Although clinically significant bowel obstruction due to diaphragm disease is uncommon, it is a potential life-threatening complication of NSAID use. Clinicians should take a detailed history of NSAID use in every patient presenting with subacute bowel obstruction. It is also important to recognise that NSAID use results in a spectrum of complications as we saw in this patient who experienced duodenal ulceration leading to perforation and clinically significant diaphragm disease.
